# Nontraumatic Exertional Rhabdomyolysis Leading to Acute Kidney Injury in a Sickle Trait Positive Individual on Renal Biopsy

**DOI:** 10.1155/2018/5841216

**Published:** 2018-04-15

**Authors:** Kalyana C. Janga, Sheldon Greenberg, Phone Oo, Kavita Sharma, Umair Ahmed

**Affiliations:** ^1^Department of Nephrology, Maimonides Medical Center, Brooklyn, NY, USA; ^2^Department of Infectious Diseases, Maimonides Medical Center, Brooklyn, NY, USA

## Abstract

A 26-year-old African American male with a history of congenital cerebral palsy, sickle cell trait, and intellectual disability presented with abdominal pain that started four hours prior to the hospital visit. The patient denied fever, chills, diarrhea, or any localized trauma. The patient was at a party at his community center last evening and danced for 2 hours, physically exerting himself more than usual. Labs revealed blood urea nitrogen (BUN) level of 41 mg/dL and creatinine (Cr) of 2.8 mg/dL which later increased to 4.2 mg/dL while still in the emergency room. Urinalysis revealed hematuria with RBC > 50 on high power field. Imaging of the abdomen revealed no acute findings for abdominal pain. With fractional excretion of sodium (FeNa) > 3%, findings suggested nonoliguric acute tubular necrosis. Over the next couple of days, symptoms of dyspepsia resolved; however, BUN/Cr continued to rise to a maximum of 122/14 mg/dL. With these findings, along with stable electrolytes, urine output matching the intake, and prior use of proton pump inhibitors, medical decision was altered for the possibility of acute interstitial nephritis. Steroids were subsequently started and biopsy was taken. Biopsy revealed heavy deposits of myoglobin. Creatinine phosphokinase (CPK) levels drawn ten days later after the admission were found to be elevated at 334 U/dl, presuming the levels would have been much higher during admission. This favored a diagnosis of acute kidney injury (AKI) secondary to exertional rhabdomyolysis. We here describe a case of nontraumatic exertional rhabdomyolysis in a sickle cell trait (SCT) individual that was missed due to findings of microscopic hematuria masking underlying myoglobinuria and fractional excretion of sodium > 3%. As opposed to other causes of ATN, rhabdomyolysis often causes FeNa < 1%. The elevated fractional excretion of sodium in this patient was possibly due to the underlying inability of SCT positive individuals to reabsorb sodium/water and concentrate their urine. Additionally, because of their inability to concentrate urine, SCT positive individuals are prone to intravascular depletion leading to renal failure as seen in this patient. Disease was managed with continuing hydration and tapering steroids. Kidney function improved and the patient was discharged with a creatinine of 3 mg/dL. A month later, renal indices were completely normal with persistence of microscopic hematuria from SCT.

## 1. Introduction

Rhabdomyolysis is a condition characterized by muscle cell death and the release of muscle cell constituents into the circulation. The causes of rhabdomyolysis include trauma +/− muscle compression; nontraumatic, nonexertional causes (drugs, toxins, or infections); and nontraumatic, exertional rhabdomyolysis [1]. The incidence of exertional rhabdomyolysis is unknown; however, a recent retrospective cohort study revealed that, out of all their rhabdomyolysis cases in a set amount of time, 35% were exertional [2]. Nontraumatic, exertional rhabdomyolysis can occur in extreme exertion or normal physical exertion in addition to risk factors that impair muscle oxygenation, ultimately leading to muscle cell death. One of these risk factors includes individuals with the sickle cell trait (SCT).

Sickle cell trait is the heterozygous state (HBAS) of sickle cell disease [3]. SCT is a benign carrier state; it is by itself not considered a disease [4]. SCT is present in 7–9% of the African American population. Additionally, a recent cross-sectional study reviewing hemoglobin phenotypes in African Americans with end stage renal disease found that SCT was twice as common among African Americans with end stage renal disease [5]. Although the overall effects of SCT are benign, many studies and case reports have identified that individuals with SCT are at an increased risk for rare conditions including exertional rhabdomyolysis with prolonged physical activity, compartment syndrome, and sudden cardiac death [6]. We report on an SCT patient with symptoms of hematuria and isosthenuria developing stage III nonoliguric AKI from exertional rhabdomyolysis.

## 2. Case Presentation

A 26-year-old African American male with a history of congenital cerebral palsy, sickle cell trait, and intellectual disability presented with abdominal pain that started four hours prior to the hospital visit. His only medication is occasional proton pump inhibitors for indigestion and belching. Abdominal pain was characterized as colicky, constant, and located in the epigastric region and was associated with symptoms including nausea and two episodes of vomiting. The patient denied fever, chills, diarrhea, or any localized trauma. The patient was at a party at his community center last evening and danced for 2 hours, physically exerting himself more than usual. Physical examination revealed mild epigastric tenderness upon palpation; skin was warm and dry with normal turgor. The buccal mucosa was moist. Blood work revealed BUN of 41 mg/dL (reference range: 7–18 mg/dL) and creatinine of 2.8 mg/dL (reference range: 0.6–1.2 mg/dL) which later increased to 4.2 mg/dL while still in the emergency room. Additionally, liver enzymes were minimally elevated. Urinalysis revealed microscopic hematuria with RBC > 50 on high power field. Imaging of the abdomen was benign. Decision was made to admit the patient based on abnormal liver enzymes and worsening kidney injury suggesting signs of nonoliguric acute tubular necrosis.

Over the next couple of days, symptoms of dyspepsia resolved; however, renal indices continued to worsen despite adequate hydration and 1 ml/kg/h bicarbonate infusion, reaching a peak of creatinine of 14 mg/dL. Due to these findings, stable electrolytes, urine output matching the intake, and prior use of occasional proton pump inhibitors, medical decision was altered for the possibility of allergic interstitial nephritis. Steroids were subsequently started and biopsy was taken.

**Figure 1:**
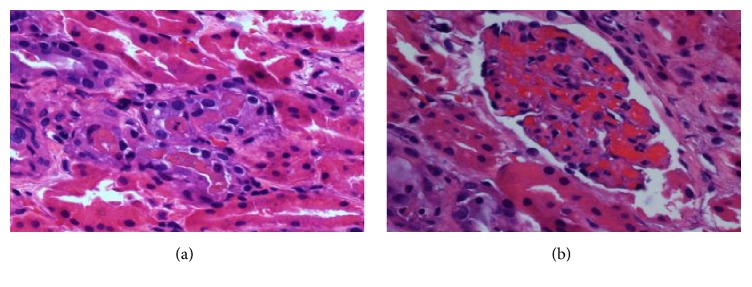
H&E stain. (a) Reddish tubular casts. Most tubules are preserved, mild interstitial fibrosis with tubular atrophy. (b) Glomeruli with congestion.

**Figure 2:**
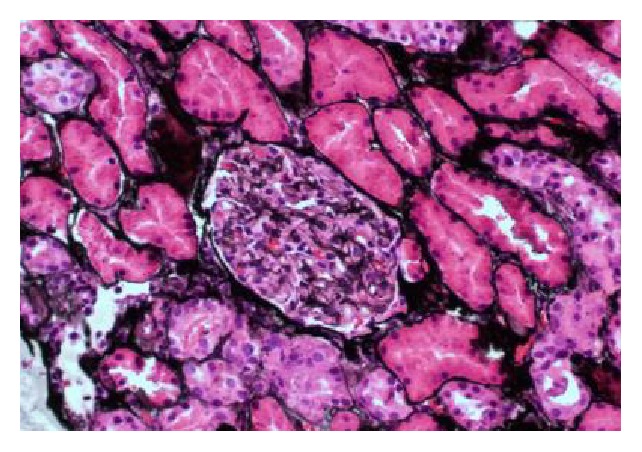
PAS stain. PAS stain showing tubular atrophy, glomeruli congestion, and normal capillary loops in the glomerulus.

**Figure 3:**
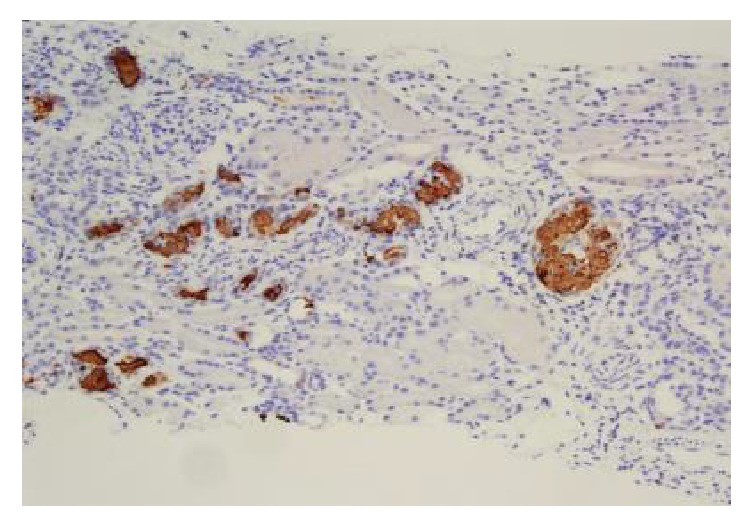
Tubular casts are positive for myoglobin immunostain.

Biopsy revealed heavy deposits of myoglobin (Figures 1–3). CPK levels were drawn shortly after the results (day 10) and were found to be elevated at 334 U/L. This favored a diagnosis of AKI secondary to exertional rhabdomyolysis. Decision was made to continue hydration and taper steroids. Kidney function improved and the patient was discharged with a creatinine of 3 mg/dL. A month later, renal indices were completely normal with persistence of microscopic hematuria from SCT.

## 3. Discussion

In this case, the patient initially presented with symptoms of nonoliguric acute tubular necrosis (ATN). The patient had abdominal pain, elevated liver enzymes, elevated BUN/Cr, and FeNa > 3%. Intrarenal AKI is characterized as damage to the major structures of the kidney including the tubules, glomeruli, interstitium, and the intrarenal blood vessels [7]. Damage can be caused by toxins, contrast, drugs, myoglobin, and others (Table 1). Although the patient's symptoms resolved the next day, his BUN/Cr continued to rise with hydration. Medical decision was altered for the possibility of proton pump inhibitor induced acute interstitial nephritis. So, steroids were started and biopsy was taken, which revealed rhabdomyolysis.

Rhabdomyolysis is a condition characterized by muscle cell death and the release of muscle cell constituents into the circulation. Common symptoms of rhabdomyolysis include muscle pain, rashes, weakness, and dark colored urine due to myoglobinuria [8, 9]. Additional symptoms in severe cases include fever, altered mental status, nausea/vomiting, or abdominal pain which our patient had. Physical examination findings may include positive muscle tenderness, muscle swelling, and/or skin discolorations. Laboratory diagnosis is essential based on the measurement of biomarkers of muscle injury, with creatinine phosphokinase being the biochemical “gold standard” for diagnosis and myoglobin the “gold standard” for prognostication, especially in patients with nontraumatic rhabdomyolysis. Serum CPK levels are usually at least 5 times the upper limit of normal at presentation, reaching the peak within 24–72 hours and then declining after 3–5 days with cessation of muscle injury [9]. The CPK levels are more sustained and long-lasting as compared to myoglobin which has a short half-life due to faster elimination kinetics. Most believe that CPK levels correlate with the amount of muscle injury and disease activity. Additional tests such as muscle biopsy, kidney biopsy, MRI, and electromyography are usually not needed [8, 9]. The filtered myoglobin is degraded into a heme pigment which can damage the kidney in three different ways, that is, direct tubular cell injury, vasoconstriction leading to decreased blood flow, and tubular obstruction [10]. However, in contrast to other causes of intrarenal AKI, the fractional excretion of sodium is often <1% and urine sodium is <20. This is possibly due to the vasoconstriction and volume depletion often observed in rhabdomyolysis. Urinalysis may also reveal findings of myoglobinuria, a positive dipstick with the absence of RBCs on urine microscopy.

It may be difficult to differentiate between hematuria and myoglobinuria. Both present with red-brown colored urine and a positive urine reagent strip test. This test is able to detect heme-positive compounds including hemoglobin and myoglobin because heme catalyzes the oxidation of tetramethylbenzidine which produces a color change [11]. The sensitivity of the test decreases with elevated specific gravity or high urinary protein. A microscopic evaluation must be done to detect the presence of RBCs and differentiate between hematuria and myoglobinuria [8, 9]. Other tests to detect myoglobin include qualitative tests such as the precipitation test and capillary electrophoresis [11]. Our patient did have a positive urine dipstick with large amounts of hemoglobin found on microscopic evaluation. This suggested the symptoms of hematuria commonly found in SCT positive patients, rather than myoglobin from rhabdomyolysis.

As discussed above, the causes of rhabdomyolysis include nontraumatic, exertional rhabdomyolysis. Patients may develop exertional rhabdomyolysis with no underlying disease and normal muscle tissue. A recent case report has shown an increasing number of patients diagnosed with exertional rhabdomyolysis after attending a spinning class [12]. None of these patients were on medications, nor did they have any significant past medical history. Although not a prerequisite, most patients that do develop exertional rhabdomyolysis will have some underlying disease or risk factors that impair muscle oxygenation, ultimately leading to muscle cell death. One of these risk factors includes individuals with the sickle cell trait (SCT).

SCT is heterozygous for the sickle hemoglobin (HbS) point mutation and is considered a benign carrier state [4]. Most individuals with SCT are asymptomatic; however, patients may develop symptoms of isosthenuria or episodes of hematuria. Isosthenuria is the inability to concentrate the urine, resulting in symptoms of polyuria, enuresis, and higher incidences of dehydration. Patients may also experience episodes of hematuria spontaneously or with heavy physical exertion. The pathophysiology behind this is due to the microvascular obstruction by rigid erythrocytes, which will lead to tissue necrosis and renal papillary infarcts causing the hematuria [13, 14]. Many studies have identified that patients with SCT are at an increased risk of exertional rhabdomyolysis. It was found that there was a significantly higher risk of exertional rhabdomyolysis in African American US military army soldiers with SCT. Additionally, similar increased risks were observed in obese and tobacco using individuals [15]. In fact, due to the increasing number of published case reports of sudden death and exertional rhabdomyolysis occurring at a high rate in SCT positive military personnel, the Department of Defense developed screening guidelines in 1972 for the testing of SCT in new recruits [16]. The increased risk of exertional rhabdomyolysis found in SCT individuals may be due to the underlying renovascular changes combined with the inability to concentrate the urine. The pathophysiology behind this is that RBC sickling found in SCT may cause the congestion of the vasa recta. This may lead to ischemia and retention of the medullary interstitium. Since water reabsorption is dependent on interstitial osmolality, there will be a decrease of reabsorption across antidiuretic hormone stimulated collecting ducts [3]. With the inability to concentrate the urine, SCT positive individuals are more inclined to dehydration and are unable flush out renal toxins adequately.

Our patient had nontraumatic exertional rhabdomyolysis due to excessive physical exertion the night before his symptoms began. As discussed above, he was at an increased risk of developing this condition because he had the sickle cell trait. The diagnosis of rhabdomyolysis was missed because of symptoms of hematuria, FeNa > 3%, and failure to order CPK levels initially. Although he received the proper treatment of fluid/electrolyte replacement, his condition took longer to recover because of the underlying renal disease found in SCT patients. Fortunately, this patient was able to recover and his kidney function normalized without the need for hemodialysis. Acute exertional rhabdomyolysis may lead to metabolic acidosis and electrolyte disturbances including hyperkalemia [17]. Severe cases may lead to renal failure and death. Although continued renal replacement therapy and hemodialysis will improve creatinine, BUN, and potassium and reduce the duration of oliguria phase and length of hospital stay, no significant differences were found in mortality rates compared with conventional therapy in rhabdomyolysis. It is important to build awareness and counsel SCT positive patients and families on avoiding excessive physical exertion in order to avoid such complications.

## 4. Conclusion

This case report describes the occurrence of nontraumatic exertional rhabdomyolysis in individuals that have the sickle cell trait. There is an increased risk of AKI secondary to exertional rhabdomyolysis in these individuals. The rhabdomyolysis was missed here due to no history of trauma, microscopic hematuria, and fractional excretion of sodium > 3%. Classic rhabdomyolysis subjects will have some type of trauma, no microscopic hematuria, and fractional excretion of sodium < 1%. In summary, we recommend checking CPK levels in all unknown causes of acute renal failure and renal biopsy should remain the gold standard in determining the cause of unknown AKI (Table 2). We also recommend screening of African American patients for SCT due to the high prevalence in this population. Early screening as done in military recruits will allow for proper counseling of these individuals in order to prevent complications including exertional rhabdomyolysis, which, through multiple published case reports, have proved to be detrimental in some patients. It is important for physicians to counsel SCT patients on these increased risks of kidney injury and advise them to avoid nephrotoxins, stay hydrated, and avoid excessive physical exertion.

## Figures and Tables

**Table 1:** Basic investigation for acute renal failure (^*∗*^tests can be ordered if needed based on history and physical).

Blood tests	Complete blood counts with differentials
	Complete metabolic profile
	Phosphorus
	Uric acid
	Myoglobin
	Creatinine phosphokinase
	Liver function tests
	Brain natriuretic peptide^*∗*^ (BNP)
	Arterial blood gas

Urine tests	Urinalysis with microscopy and culture
	Urine osmolality
	Urine electrolytes
	Urine eosinophils^*∗*^
	Urine protein/creatinine ratio (PCR)

Radiology tests	Renal and bladder sonogram
	Chest X-ray^*∗*^

**Table 2:** Renal biopsy indications.

Isolated glomerular hematuria	Persistent and severe hematuria, hypertension, and elevated creatinine

Isolated nonnephrotic syndrome	Persistent and >1-gram proteinuria

Nephrotic syndrome	Routinely indicated in adults including diabetes, connective tissue disorders, steroid-resistant nephrotic syndrome, and obesity Exception is first attack in children

Nephritic syndrome	Connective tissue disorders, hepatitis, infection-related nephritic syndrome, and rapidly progressive glomerulonephritis

Unexplained acute kidney injury	After clinical diagnosis fails

Unexplained progressive chronic kidney disease	Normal size kidneys and extent of kidney damage

Familial renal diseases	Biopsy of one member can yield the diagnosis of other family members

Renal transplant dysfunction	Acute versus chronic rejection versus drug-induced renal failure

Small localized renal tumors/lesions	Benign renal tumors, metastasis, lymphoma, and focal kidney infection
